# Ti Porous Film-Supported NiCo_2_S_4_ Nanotubes Counter Electrode for Quantum-Dot-Sensitized Solar Cells

**DOI:** 10.3390/nano8040251

**Published:** 2018-04-17

**Authors:** Jianping Deng, Minqiang Wang, Xiaohui Song, Zhi Yang, Zhaolin Yuan

**Affiliations:** 1 Shaanxi Key Laboratory of Industrial Automation, School of Physics and Telecommunication Engineering, Shaanxi University of Technology, Hanzhong 723001, China; yuanzhaolin98@126.com; 2 Electronic Materials Research Laboratory, Key Laboratory of the Ministry of Education & International Center for Dielectric Research, School of Electronic and Information Engineering, Xi’an Jiaotong University, Xi’an 710049, China; yangzhi029@xjtu.edu.cn; 3 Henan Key Laboratory of Photovoltaic Materials, College of Physics and Materials Science, Henan Normal University, Xinxiang 453000, China; songsxh@126.com

**Keywords:** NiCo_2_S_4_ nanotubes, Ti porous film, quantum dot, solar cells, counter electrode

## Abstract

In this paper, a novel Ti porous film-supported NiCo_2_S_4_ nanotube was fabricated by the acid etching and two-step hydrothermal method and then used as a counter electrode in a CdS/CdSe quantum-dot-sensitized solar cell. Measurements of the cyclic voltammetry, Tafel polarization curves, and electrochemical impedance spectroscopy of the symmetric cells revealed that compared with the conventional FTO (fluorine doped tin oxide)/Pt counter electrode, Ti porous film-supported NiCo_2_S_4_ nanotubes counter electrode exhibited greater electrocatalytic activity toward polysulfide electrolyte and lower charge-transfer resistance at the interface between electrolyte and counter electrode, which remarkably improved the fill factor, short-circuit current density, and power conversion efficiency of the quantum-dot-sensitized solar cell. Under illumination of one sun (100 mW/cm^2^), the quantum-dot-sensitized solar cell based on Ti porous film-supported NiCo_2_S_4_ nanotubes counter electrode achieved a power conversion efficiency of 3.14%, which is superior to the cell based on FTO/Pt counter electrode (1.3%).

## 1. Introduction

In recent years, the quantum-dot-sensitized solar cell (QDSSC) has aroused a widespread attention due to the large absorption coefficient, multiple exciton generation, and the tunable absorption spectrum based on quantum confinement effect. QDSSC is composed of three parts: QD-sensitized TiO_2_ or ZnO photoanode, electrolyte (S_n_^2−^/S^2−^), and counter electrode (CE) [1]. As one of the most important parts in QDSSC, CE is used as a catalyst to reduce S_n_^2−^/S^2−^ after the electron injection from external circuit so that QD can be regenerated. For achieving this function, CE materials should provide superior catalytic activity and high chemical stability against the corrosive polysulfide electrolyte.

As is well known, Pt is a poor electrocatalyst for reducing S_n_^2−^/S^2−^ due to its strong chemisorption with S^2−^ ions, resulting in the serious corrosion and much higher overpotentials for electrolyte regeneration. Therefore, the QDSSC with Pt CE show a low fill factor (FF) and power conversion efficiency (PCE) [2], and the high cost is another disadvantage. Recently, some Pt-free CE materials with low cost, such as carbon materials [3,4,5], conductive polymers [6], and inorganic compound [7], have been widely developed and demonstrated to have attractive performances. Of these Pt-free CE materials, the transition-metal sulfides, such as CuS, FeS, CoS, NiS, and NiCo_2_S_4_ [8,9,10,11,12], have attracted tremendous interest. Especially, NiCo_2_S_4_ contains higher electrochemical characteristics compared with binary NiS and CoS. NiCo_2_S_4_ has been regarded as one of the most potential electrode materials for a super-capacitor for are several main reasons, as follows: (1) NiCo_2_S_4_ has a high electric conductivity, which is approximately 100 times higher than that of NiCo_2_O_4_ and higher than that of NiS and CoS [13]; (2) NiCo_2_S_4_ has good mechanical and thermal stability and two different metal cations (Co and Ni) supplying richer redox reactions, leading to better electrochemical performance [14,15,16,17]. Up until now, NiCo_2_S_4_ as an efficient CE has also been widely used in dye-sensitized solar cell (DSSC). Shi et al. reported that NiCo_2_S_4_ nanosheet films were used as a CE of DSSC, the photocurrent density is increased by 3 mA/cm^2^ [18]. Huo et al. fabricated the flower-like NiCo_2_S_4_/NiS micro-spheres, then the NiCo_2_S_4_/NiS was coated on FTO (fluorine doped tin oxide) conductive glass as a CE for DSSC and the PCE of DSSC increased by 8.24% compared with that of the DSSC based on Pt CE [19]. A compact NiCo_2_S_4_ film with a thickness of 40 nm and a cross-linked network of NiCo_2_S_4_ nanosheet film coated FTO conductive glass were used as CEs for DSSC [20,21], and the DSSC with NiCo_2_S_4_ CE exhibited higher PCE compared with that of Pt CE-based DSSC. In addition, one-dimensional (1D) nanomaterials (e.g., nanorod, nanowire, nanotube) with direct electrical pathways show excellent application prospects in nanoscale electronic devices. One-dimensional NiCo_2_S_4_ nanotube arrays were used for supercapacitors [22] and 1D Co_9_S_8 _hollow nanoneedle arrays were used as CE for QDSSC [11].

The substrate supporting CE materials is also very important for the performance of DSSC; it should have large surface area, excellent conductivity, and good corrosion resistance to the electrolyte. Owing to the poor conductivity of metal sulfides, improving the catalytic activity of CE by increasing the thickness of CE is limited. In order to solve this problem, the porous microstructure with a large surface is used to load CE materials. For example, porous SnO_2_, ZnO, TiO_2_, and carbon and nickel foam were used as catalyst support [7,9,23,24]. The porous Cu_2_S and FeS CEs were directly prepared on Cu and Fe substrates by in situ corrosion method [25], but they can easily peeled off from the substrates because of the reaction of Cu and Fe substrates with S^2−^ in electrolyte. In addition, it is important that the electrons from the external circuit quickly transfer to CE materials and reduce the electrolyte. So far, various substrates have been used, such as FTO and ITO (indium doped tin oxide) conductive glass [3,4,5,6], C fiber cloth and C paper [16], Ti mesh [26], and abovementioned Fe and Cu. Generally, two methods are available to prepare CE: one is the in situ growth method, the other is the ex situ coating method. For the former, there is a good adhesion between the substrate and CE materials, but the load of CE materials is limited. For the latter, although CE materials can be increased by increasing the coating several times, the adhesion is poor. In order to solve the adhesion problem, the adhesive was added into the CE materials [27], but it increased the electron transfer resistance.

In this paper, we have designed NiCo_2_S_4_ nanotubes supported on Ti porous film (Ti-PF) as CE for QDSSC. Firstly, Ti-PF was prepared by acid etching, then NiCo_2_S_4 _nanotubes were synthesized on Ti-PF by two-step hydrothermal method. NiCo_2_S_4_ nanotubes not only provide the effective path for electron transport but also have more electroactive sites for reducing polysulfide electrolyte. In addition, Ti-PF/NiCo_2_S_4_ CE exhibits lower charge-transfer resistance compared with FTO/Pt CE owing to the high conductivity of Ti and the porous structure increases the load of CE materials and improves the stability via the pore-wall. As a result, the PCE (3.14%) of QDSSC based on Ti-PF/NiCo_2_S_4_ CE is higher than that (1.3%) of QDSSC based on Pt CE.

## 2. Experimental Section

**Figure 1:**
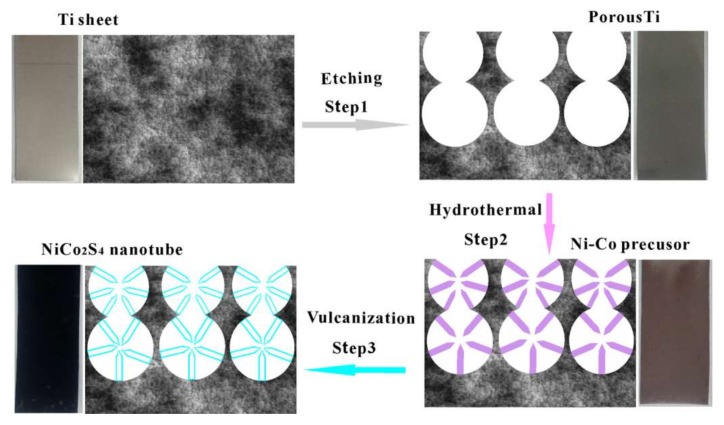
Schematic diagram to illustrate the preparation process of NiCo_2_S_4_ nanotubes on Ti porous film (Ti-PF).

In this work, NiCo_2_S_4_ nanotubes supported on Ti-PF were prepared by the following three steps (Figure 1): (1) Ti-PF was prepared by acid etching as the substrates (Step 1); (2) Ni−Co precursor ((Ni,Co)_2_(CO_3_)(OH)_2_) nanorods were hydrothermally grown (Step 2); and (3) (Ni,Co)_2_(CO_3_)(OH)_2_ nanorods were converted into NiCo_2_S_4_ nanotubes in Na_2_S solution via an anion-exchange reaction (Step 3).

### 2.1. Preparation of Ti-PF

Firstly, Ti sheets with high purity (TA1, 99.9%) and 0.2 mm thickness were washed in the acetone and ethanol using an ultrasonic bath for 30 min, respectively, and rinsed with deionized water. Then, the cleaned Ti sheets were immersed in 90 mL of HCl solution (25 wt·%) for 24–72 h at room temperature. Next, Ti-PF sheets were washed thoroughly with deionized water until the pH was close to 7 and further dried in air.

### 2.2. Fabrication of NiCo_2_S_4 _Nanotubes

NiCo_2_S_4 _nanotubes were prepared by two-step hydrothermal method according to the literature [14,28]. All the reagents were of analytical grade in this experiment and purchased from Sinopharm (Beijing, China). Firstly, 4 mmol CoCl_2_·6H_2_O, 2 mmol NiCl_2_·6H_2_O, and 12 mmol urea were dissolved in 35 mL deionized water and stirred to form a pink homogeneous solution. Subsequently, the mixed-solution and Ti-PF sheets were transferred into 50 mL Teflon-lined stainless-steel autoclave and then heated at 120 °C for 10 h. After being cooled to room temperature, Ti-PF sheets with pink product were washed with deionized water and ethanol and then dried at 60 °C in air for 10 h. The (Ni,Co)_2_(CO_3_)(OH)_2_ nanorods were obtained. In the next step, the (Ni,Co)_2_(CO_3_)(OH)_2 _was transformed into NiCo_2_S_4_ by a hydrothermal process in 0.1 M Na_2_S·9H_2_O solution at 120 °C for 10 h. After being cooled to room temperature, Ti-PF sheets with black products were washed with deionized water and ethanol and then dried in air at 60 °C. The NiCo_2_S_4_ nanotube CE was obtained.

### 2.3. Assembly of QDSSCs

The QDSSC was fabricated using a screen printing technique with the home-made TiO_2_/ZnO paste [29]. Firstly, the TiO_2_ compact layer was prepared via a spin-coating method and followed by calcination at 400 °C for 0.5 h. Subsequently, the mesoscopic photoanodes were prepared through four circulars screen printing of TiO_2_/ZnO paste on FTO conductive glass with TiO_2_ compact layer and sintered at 450 °C for 0.5 h. The active area of the film is 0.25 cm^2^. Next, the growth of ZnO nanowires was performed in a procedure similar to that in our previous paper. Lastly, CdS/CdSe/ZnS QDs were deposited by successive ionic layer adsorption and reaction (SILAR) method [1,29]. A polysulfide electrolyte used in the QDSSCs and the symmetric cells was prepared by dissolving 1 M Na_2_S, 1 M S and 0.2 M KCl in a methanol/water solution (7:3, *v*/*v*).

### 2.4. Characterization and Measurements

Field emission scanning electron microscopy (FE-SEM, S-4800, Hitachi, Tokyo, Japan) and transmission electron microscope (TEM, Tecnai G2F20, FEI, Columbus, OH, USA) were carried out to investigate the morphology and composition. The X-ray diffraction (XRD) patterns were obtained by D/max-2400 X-ray diffraction spectrometer (Rigaku, Akishima-Shi, Japan) with Cu Ka radiation at 40 kV and 100 mA. The current-voltage (I–V) characterization was performed under AM 1.5 G simulated sunlight (100 mW/cm^2^) and recorded by a Keithley 2400 Source Meter (Keithley Instruments, Inc., Cleveland, OH, USA). The cyclic voltammetry (CV), Tafel polarization curves, and electrochemical impedance spectroscopy (EIS) were performed in the symmetric cells on the workstation (CHI660E, CH Instruments Ins., Shanghai, China). These tests were used to investigate the electrocatalytic ability of CE towards the reduction of S_n_^2−^/S^2−^ electrolyte and the electronic transport properties of CE. In CV tests, the scanning potential range is from −1.2 V to 1 V with a scan rate of 100 mV/s. EIS curves were recorded at bias voltage of 0 V over a frequency range of 0.1 Hz to 1 MHz with AC amplitude of 10 mV, all EIS spectra were analyzed by ZsimpWin software. Polarization Tafel curves were recorded from −0.6V to 0.6 V at the scan rate of 10 mV/s.

## 3. Results and Discussion

### 3.1. Morphology of Ti-PF

**Figure 2:**
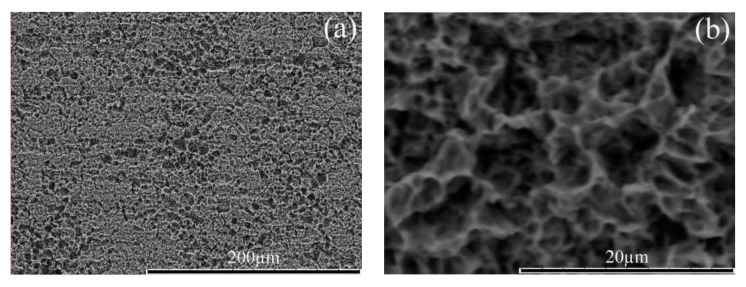

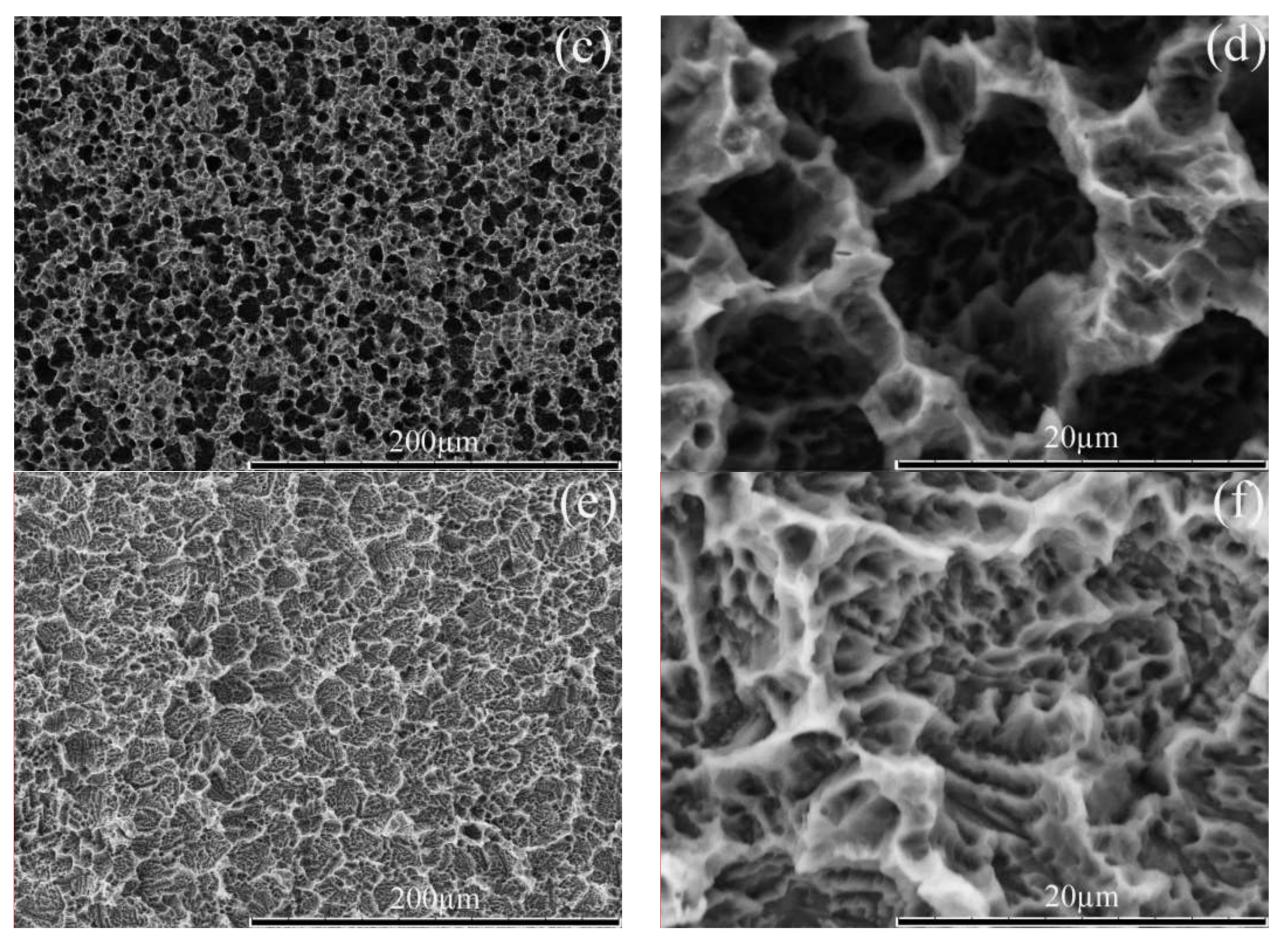
SEM images for Ti-PF from different etching time. (**a**,**b**)24 h, (**c**,**d**)48 h, and (**e**,**f** )72 h.

Figure 2 shows the SEM images for Ti-PF with different etching time in HCl. Generally, metal Ti is stable in low concentration of HCl at room temperature. However, Ti is slowly etched when HCl concentration is greater than 20%. In our experiment, HCl solution with concentration of 25% is used to etch Ti sheets at room temperature and the morphology of Ti-PF is controlled by adjusting the etching time. Figure 2a–f show the morphologies of Ti-PF with etching 24 h, 48 h, and 72 h, respectively. It can be seen clearly that with the increase of etching time, the porous structure has changed significantly. When the etching time is 24 h, the holes with the size range of about 5–10 μm exist only in very few places (Figure 2a,b). When the etching time increases to 48 h, the holes with the size range of 10–20 μm are uniformly formed on the surface of Ti sheet (Figure 2c,d). With an increase in etching time (up to 72 h), the holes disappeared completely (Figure 2e) and the shallow pits with the size of below 5 μm were formed (Figure 2f). In this experiment, Ti-PF plays three roles to improve the catalytic activity of CE to polysulfide electrolyte: (1) metal Ti provides a fast electronic transmission channel, (2) the porous structure gives a large surface area and thus increases the load of CE materials, and (3) the deeper holes in which NiCo_2_S_4_ nanotubes are limited by the wall increase the stability of CE. Therefore, the Ti-PF etched for 48 h is most suitable for using as the CE substrate.

### 3.2. Morphology of NiCo_2_S_4_ Nanotubes

The conversion of (Ni,Co)_2_(CO_3_)(OH)_2_ nanorods into NiCo_2_S_4_ nanotubes can be explained by the anion-exchange reaction mechanism [22,30,31]. Firstly, S^2-^ in the Na_2_S solution exchanges with CO_3_^2−^ and OH^−^ on the surface of (Ni, Co)_2_(CO_3_)(OH)_2 _nanorods to form NiCo_2_S_4_, CO_3_^2−^, and OH^-^ react with H^+^ in the solution to produce CO_2_ and H_2_O. At the same time, the internal (Ni,Co)_2_(CO_3_)(OH)_2_ diffuse spontaneously to the surface of the nanorod, where it provides a source of (Ni,Co)_2_(CO_3_)(OH)_2_ for further anion exchange. The continuous outward diffusion results in the generation of void space inside the nanorod. When (Ni,Co)_2_(CO_3_)(OH)_2_ has been completely converted into NiCo_2_S_4_, nanorods become nanotubes.

**Figure 3:**
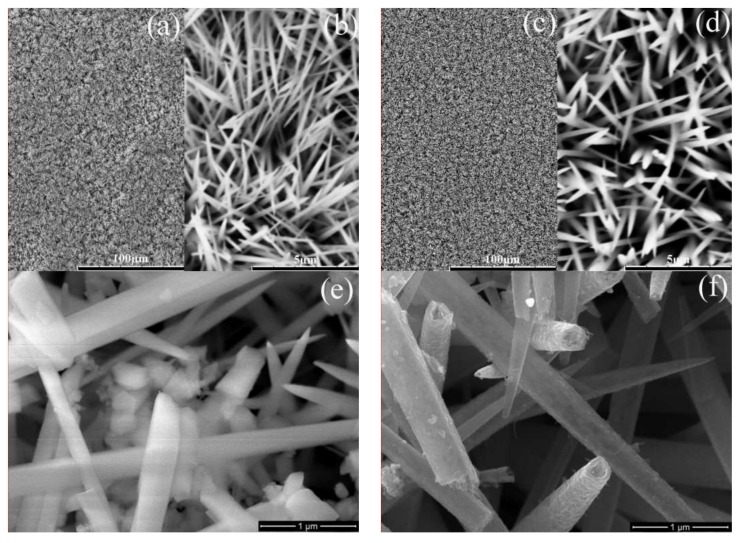
SEM images for (Ni,Co)_2_(CO_3_)(OH)_2_ nanorods (**a**,**b**,**e**) and NiCo_2_S_4_ nanotubes (**c**,**d**,**f**) on Ti-PF.

To illustrate the morphology of as-synthesized samples, SEM measurements were performed. Figure 3a,b,e show the representative low-magnification and high-magnification SEM images of (Ni,Co)_2_(CO_3_)(OH)_2_ nanorods, respectively. Figure 3c,b,f correspond to the low-magnification and high-magnification SEM images of NiCo_2_S_4 _nanotubes, respectively. In Figure 3a–d, (Ni,Co)_2_(CO_3_)(OH)_2_ nanorods and NiCo_2_S_4_ nanotubes were homogeneously deposited on Ti-PF, suggesting that the two-hydrothermal method is favorable in forming this structure. Moreover, the NiCo_2_S_4 _nanotubes were well preserved during sulfurization process. It can be seen that the (Ni,Co)_2_(CO_3_)(OH)_2_ array film is composed by many multi-directional nanorods due to the nucleation sites from the wall of holes and there is considerable inter-nanorod space, it will help electrolyte full contact with CE materials at the bottom, improving the utilization rate of the CE materials. A rough comparison between Figure 3b,d the diameters of NiCo_2_S_4_ nanotubes are larger than that of (Ni,Co)_2_(CO_3_)(OH)_2_ nanorods, owing to the Ni and Co ions diffusion from inside to outside of the nanorod during an anion-exchange process. As shown in Figure 3e,f (Ni,Co)_2_(CO_3_)(OH)_2_ nanorods with a smooth surface and solid structure and NiCo_2_S_4 _nanotubes with a rough surface are clearly seen from the damaged film.

**Figure 4:**
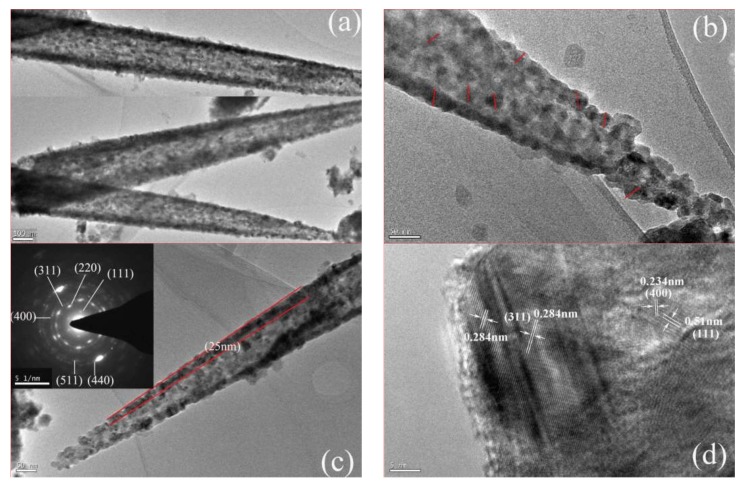
(**a**–**c**) TEM and (inset) SAED and (**d**) HRTEM images of NiCo_2_S_4_ nanotube.

The detailed structure of NiCo_2_S_4_ nanotube scraped from Ti-PF sheet was further confirmed by TEM, as shown in Figure 4. From Figure 4a, the nanotube structure and the porous wall can be evidently seen, indicating the successful preparation of NiCo_2_S_4_ nanotubes on Ti-PF. By a closer examination of the wall in Figure 4b, it is found that NiCo_2_S_4_ nanotube is composed of many nanoparticles with a size of about 25 nm (marked with red line) and numerous pores locate at the nanotube. The NiCo_2_S_4_ nanotube with a rough surface (Figure 4a–c) and a thin wall of about 25 nm (Figure 4c) effectively increases the electroactive sites. The nanotube structure can greatly enhance the electrolyte penetration and improve the performance of cells. In Figure 4c, the corresponding selected area electron diffraction (SAED) pattern indicates the polycrystalline nature of NiCo_2_S_4_ nanotubes and the diffraction rings can be readily indexed to the (111), (220), (311), (400), (511), and (440) planes of NiCo_2_S_4_ phase. In addition, Figure 4d reveals that the lattice spacings are about 0.51 nm, 0.284 nm, and 0.234 nm, which can be assigned to the (111), (311), and (400) crystal planes of the cubic NiCo_2_S_4_ phase, respectively, indicating the successful formation of crystalline NiCo_2_S_4_.

**Figure 5:**
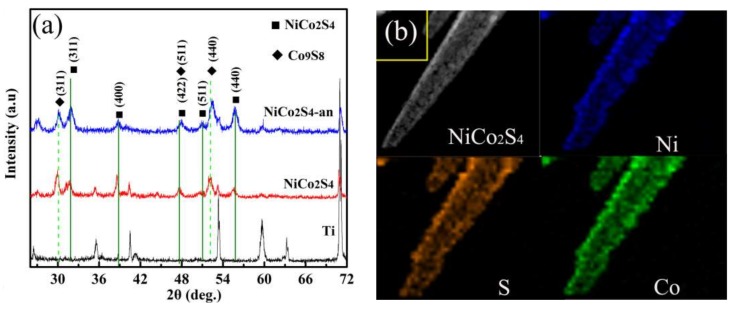
(**a**) XRD images for Ti-PF, NiCo_2_S_4_, and NiCo_2_S_4_-an on Ti-PF; (**b**) TEM image and the corresponding EELS elemental mapping images of a single NiCo_2_S_4_ nanotube.

The as-synthesized NiCo_2_S_4_ nanotubes were further confirmed by the XRD and electron energy loss spectroscopy (EELS) elemental mapping. In order to investigate the effect of annealing treatment on the catalytic activity of NiCo_2_S_4_ CE and the performance of cells, NiCo_2_S_4_ CE was annealed at 400 °C for 30 min in the nitrogen atmosphere (NiCo_2_S_4_-an). Figure 5a shows XRD images for Ti-PF, NiCo_2_S_4_, and NiCo_2_S_4_-an on Ti-PF. The diffraction peaks located at 31.6°, 38.3°, 50.5°, and 55.3° can be indexed to the (311), (400), (511), and (440) planes of the cubic phase NiCo_2_S_4_ (JCPDS 20-0782), which is consistent with SAED analysis. There are also two strong peaks at 29.84° and 52.0°, which may correspond to the (311) and (440) planes of the cubic phase Co_9_S_8_ (JCPDS no.19-0364). The existence of Co_9_S_8_ phase is because that (Ni,Co)_2_(CO_3_)(OH)_2_ nanorods were incompletely sulfurized in Na_2_S solution, which was verified by many reports [14,32]. It can also be seen that the intensity of (311) and (440) diffraction peaks of NiCo_2_S_4 _increased after annealing, indicating an increase in the crystallinity. Moreover, the TEM and EELS mapping images (Figure 5b) indicate that the elements (Ni, Co, and S) are uniformly distributed in the NiCo_2_S_4_ nanotube.

### 3.3. Electrochemical Properties of CEs

**Figure 6:**
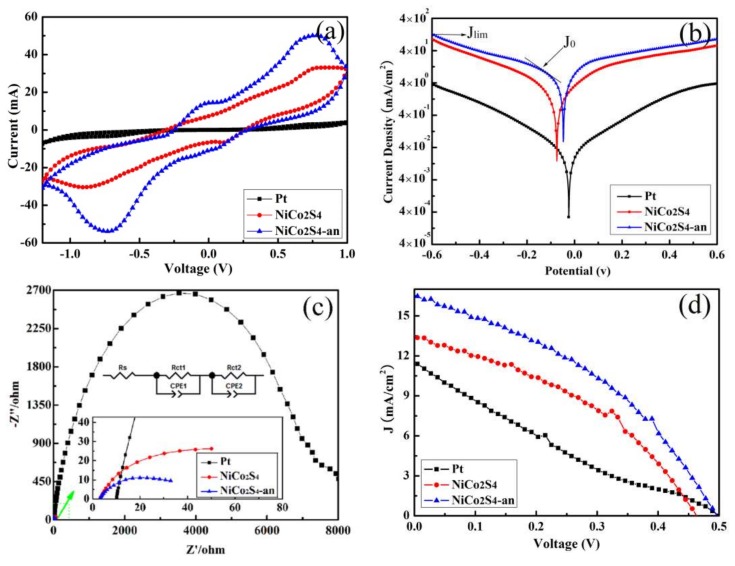
(**a**) Cyclic voltammetry (CV); (**b**) Tafel curves; and (**c**) EIS of the symmetric cells with Pt, NiCo_2_S_4_, and NiCo_2_S_4_-an CEs and (**d**) J–V characteristics for QDSSCs based on Pt, NiCo_2_S_4_, and NiCo_2_S_4_-an CEs, respectively.

To investigate the electrochemical properties of Pt, NiCo_2_S_4_, and NiCo_2_S_4_-an CEs, CV test of a symmetrical cell was carried out, as shown in Figure 6a. The peaks explain the catalytic reaction at the interface between CE and electrolyte as follows S_n_^2−^ + e^−^ → nS^2−^. From the CV curves, NiCo_2_S_4_ and NiCo_2_S_4_-an CEs present a similar shape with two typical pairs of redox peaks [16,21]. As a matter of fact, the reduction peak of the left pair is assigned to the reaction S_n_^2^^−^ + e^−^ → nS^2^^−^ and the right one is assigned as S + 2ne^−^ → S_n_^2−^ [19,20,33]. The role of the CE in a QDSSC is to catalyze the reduction of S_n_^2−^ to S^2−^ ions in the polysulfide electrolyte, so the left pair of redox peaks is directly related to the catalytic activity of CE, the positive and negative peaks correspond to the oxidation of S^2−^ and the reduction of Sn^2−^, respectively [34,35]. However, because of high over-potential, Pt CE has only one pair of redox peaks, which correspond to the oxidation of S^2−^ and the reduction of S_n_^2−^ [36]. The higher current density of left cathodic peak indicates that the CE has an excellent electrocatalytic activity for the reduction of S_n_^2−^ to nS^2−^ [17]. It can be seen that NiCo_2_S_4_ and NiCo_2_S_4_-an CEs show higher current than Pt CE and the reduction current density of NiCo_2_S_4_-an CE is the biggest. This results indicate that the NiCo_2_S_4_ CE is expected to enhance QD regeneration and photoelectron generation, thus beneficial for improving QDSSC’s photocurrent, and the annealing treatment enhanced the crystallinity of NiCo_2_S_4 _CE, which increases the reduction current.

Tafel polarization technique is an important method to evaluate the catalytic activity of CEs. Theoretically, the Tafel curve includes the diffusion, Tafel and polarization zones at the high-, middle-, and low-potential areas, respectively. In Tafel analysis, the exchange current density (*J*_0_) (Tafel zone) and the limiting diffusion current density (*J*_lim_) (diffusion zone) are two key parameters to evaluate the electrocatalytic activity of CEs. Tafel polarization curves of Pt, NiCo_2_S_4_, and NiCo_2_S_4_-an CEs are shown in Figure 6b. It can be seen that, in the Tafel zone, the slopes of the anodic or cathodic branches are in the order of NiCo_2_S_4_-an > NiCo_2_S_4_ > Pt. A larger slope indicates a higher *J*_0_. According to the following equation:

*J*_0_ = R*T*/*n*F*R*_ct_, (1)

where R, *T*, F and *n* are the gas constant, the temperature, Faraday’s constant, and the electron number involved in Sn^2−^/S^2−^ redox couple, respectively [35]; the charge-transfer resistance (*R*_ct_) can be calculated by *J*_0_ values. The change trends of the *R*_ct_ is NiCo_2_S_4_-an < NiCo_2_S_4_ < Pt, which is consistent with the EIS results. In addition, *J*_lim_ derived from the horizontal part of the curve at high potential is also closely related to the catalytic activity of CEs, which is given by equation:

*D* = *L J*_lim_/2*n*F*C*, (2)

where *D*, *L*, F, *C*, and n are the diffusion coefficient of the polysulfide, the electrolyte thickness, the Faraday constant, the polysulfide concentration, and the number of electrons involved in the reduction of disulphide at the counter electrode, respectively [37]. It can be noticed that the change trend of *J*_lim_ is consistent with CV results, suggesting the *D* of redox couple in the electrolyte increases with enhanced electrocatalytic activity of CEs.

To further understand the reason for the good performance of the as-prepared CEs, EIS was carried out using the symmetrical cells, as shown in Figure 6c, and the corresponding parameters are shown in Table 1. Figure 6c shows Nyquist plots of Pt, NiCo_2_S_4_, and NiCo_2_S_4_4-an CEs and the insets of Figure 6c are the equivalent circuit and the magnified Nyquist impedance. In the equivalent circuit, R_s _represents the series resistance including the sheet resistance of the substrates (FTO and Ti sheet) and the contact resistance of the symmetrical cell, which is mainly correlated to electron transfer rates to the interface of CE/electrolyte and it can be estimated from the intercept on the real axis at the high frequency. The intercept of the middle frequency semicircle on the real axis represents *R*_ct_ at the interface between CE and electrolyte. The *R*_ct_, which is closely related to the electrocatalytic activity and the reaction kinetics of the CE, is an important parameter to determine the FF of cell [36,38,39]. Generally, because of the symmetrical structure, *R*_ct1_ and *R*_ct2_ at the two CE/electrolyte interfaces are equal (*R*_ct1_ = *R*_ct2_), so every Nyquist plot has one semicircle [35,40]. The obtained impedance spectra are fitted by Z-View software, as shown in Table 1. The *R*_s_ values of Pt, NiCo_2_S_4 _and NiCo_2_S_4_-an CEs are 8.639 Ω, 3.139 Ωand 3.01 Ω, respectively. Among them, the R_s_ values of NiCo_2_S_4_, and NiCo_2_S_4_-an CEs are close, which may be ascribed to the same Ti-PF substrates. The *R*_s_ of Pt CE is much higher than that of NiCo_2_S_4 _and NiCo_2_S_4_-an CEs, which is attributed to the strong chemisorption of S^2−^ ions on Pt. *R*_ct_ directly reflects the electrochemical reaction at CE/electrolyte interface, *R*_ct_ values of Pt, NiCo_2_S_4_, and NiCo_2_S_4_-an CEs are 6860 Ω, 67.47 Ω, and 33.31 Ω, respectively; this means that it is easier for charges transfer through the NiCo_2_S_4_-an/electrolyte interface than Pt/electrolyte and NiCo_2_S_4_/electrolyte interfaces. Thus, it is anticipated that the QDSSC with Ti-PF-supported NiCo_2_S_4_ nanotube CE will show better photovoltaic performance. Furthermore, the proper annealing treatment reduced the *R*_ct_ of NiCo_2_S_4_/electrolyte interface and improved the short-circuit photocurrent density (*J*_sc_) and PCE of QDSSC.

Complete photovoltaic cells based on NiCo_2_S_4_ and NiCo_2_S_4_–an CEs were fabricated and the cell based on Pt CE is used as the reference. In this study, Pt, NiCo_2_S_4_, and NiCo_2_S_4_-a CEs were soaked in S_n_^2−^/S^2−^ electrolyte for 24 h and then were used in the complete photovoltaic cells. The complete photovoltaic cells and symmetric cells were fixed by clip and spacer with 90 µm thickness. The photovoltaic curves and the photovoltaic parameters (open-circuit voltage (*V*_oc_),* J*_sc_, FF, and PCE) are shown in Figure 6d and Table 1, respectively. From Figure 6d, obviously, the performance of QDSSCs based on NiCo_2_S_4_ and NiCo_2_S_4_-an CEs are better than that of QDSSC with Pt CE.

The champion QDSSC based on Pt CE has an *V*_oc_ of 0.489V, a *J*_sc_ of 11.76 mA/cm^2^, a FF of 22.56%, and a PCE of 1.3%. The champion QDSSC with NiCo_2_S_4_ CE has a *V*_oc_ of 0.456V, a *J*_sc_ of 13.72 mA/cm^2^, a FF of 40.6%, and a PCE of 2.54%. The champion QDSSC employing NiCo_2_S_4_-an CE has a *V*_oc_ of 0.489V, a *J*_sc_ of 16.68 mA/cm^2^, a FF of 38.52%, and a PCE of 3.14%. Notably, the PCE increased from 1.3% to 3.14% when Pt CE was replaced with NiCo_2_S_4_-an CE. In addition, the average values obtained of the three best cells (up to nine) based on an optimal photoanode and three CEs are given in brackets, as shown in Table 1, and the change trends of the average values of *V*_oc_, *J*_sc_, FF, and PCE are consistent with the champion QDSSCs. This improvement in the cell performance originates from the significant increases in both *J*_sc_ and FF, which closely related to the higher electrocatalytic ability of CE. Furthermore, the QDSSC with NiCo_2_S_4_-an CE shows a higher *J*_sc_ and *V*_oc_ than that with NiCo_2_S_4_ CE and thus obtains a higher PCE. *V*_oc_ of QDSSC depends upon the difference between the quasi Fermi level of the photoanode and the redox potential of the electrolyte. The annealing treatment improved the crystallinity and the conductivity of NiCo_2_S_4_, so the fast charge transfer at CE/electrolyte interface can cause a change in the concentration gradient in the electrolyte solution, which influences the recombination rates at the photoanode/electrolyte interface and consequently the conduction band position of the photoanode. Meanwhile, the high conductivity of NiCo_2_S_4_-an CE also increased the photocurrent of cell [41]. J–V parameters are in line with the electrocatalytic ability of CEs discussed in the CV, Tafel polarization, and EIS.

## 4. Conclusions

In summary, we have prepared Ti-PF by the acid etching technique and Ti-PF supporting NiCo_2_S_4_ nanotubes via two-step hydrothermal method; furthermore, Ti-PF supporting NiCo_2_S_4_ nanotubes are used as CE in QDSSCs. The morphology of Ti-PF is affected with the etching time. When etching time is 48 h in hydrochloric acid with a weight concentration of 25% at room temperature, the holes are uniformly formed on the surface of Ti sheet, which is most suitable for use as the substrate to support CE materials. SEM, TEM, and XRD results show that the as-synthesized NiCo_2_S_4 _nanotube with porous surface is the cubic phase. Using a polysulfide electrolyte in the symmetric cells, Ti-PF/NiCo_2_S_4_ CE provided greater electrocatalytic activity (a higher reduction current density, a higher *J*_0_ and *J*_lim_) and lower internal resistance (*R*_s_ and *R*_ct_). Also, Ti-PF/NiCo_2_S_4_ was used to fabricate QDSSC, it has a higher performance (*J*_sc_ = 16.68 mA/cm^2^, *V*_oc_ = 0.489 V, FF = 38.52%, and PCE = 3.14%) than that based on FTO/Pt CE (*J*_sc_ = 11.76 mA/cm^2^, *V*_oc_ = 0.489 V, FF = 22.56%, and PCE = 1.3%).

## Figures and Tables

**Table 1:** Photovoltaic parameters obtained from J–V curves of quantum-dot-sensitized solar cells (QDSSCs) and EIS parameters of symmetric cells.

Samples	*V*_oc_ (V)	*J*_sc_ (mA/cm^2^)	FF (%)	PCE (%)	*R*_s_ (Ω)	*R*_ct_ (Ω)
Pt	0.489 (0.483)	11.76 (10.29)	22.56 (25.19)	1.30 (1.21)	8.639	6860
NiCo_2_S_4_	0.456 (0.45)	13.72 (13.56)	40.60 (40.46)	2.54 (2.51)	3.139	67.47
NiCo_2_S_4_-an	0.489 (0.478)	16.68 (15.32)	38.52 (38.61)	3.14 (2.82)	3.010	33.31
